# Resistance to Dasatinib in primary chronic lymphocytic leukemia lymphocytes involves AMPK-mediated energetic re-programming

**DOI:** 10.18632/oncotarget.1508

**Published:** 2013-11-07

**Authors:** Veronica Martinez Marignac, Sarah Smith, Nader Toban, Miguel Bazile, Raquel Aloyz

**Affiliations:** ^1^ McGill University, Lady Davis Institute & Segal Cancer Center, Jewish General Hospital, Montreal, Canada; ^2^ Faculty of Medicine, Program in Cancer Genetics, McGill University, Montreal, Canada; ^3^ Departments of Experimental Medicine & Oncology, McGill University, Montreal, Canada

**Keywords:** Metformin, eIF4E, CLL, AMPK, Dasatinib, kinase inhibitor, metabolism, glycolysis, OXPHOS, signaling

## Abstract

Chronic lymphocytic leukemia (CLL) is the most common leukemia in adults in the western world. Although promising new therapies for this incurable disease are being tested in clinical trials, the therapeutic relevance of metabolic rewiring in chronic lymphocytic leukemia (CLL) is poorly understood. The aim of this study was to identify targetable metabolic differences in primary CLL lymphocytes by the use of Dasatinib. Dasatinib is a multi-tyrosine kinase inhibitor used to treat chronic myelogenous leukemia (CML) and is being tested in clinical trials for several cancers including CLL. This drug has been shown to be beneficial to CML patients suffering from diabetes by reducing their glucose plasma levels. In keeping with this previous observation, we report that Dasatinib induced glucose use while reducing lactate production, suggesting that this tyrosine kinase inhibitor decreases aerobic glycolysis and shifts glucose use in primary CLL lymphocytes. Our results suggest that primary CLL lymphocytes (independently of traditional prognostic factors) can be stratified in two subsets by their sensitivity to Dasatinib in vitro. Increased glucose use induced by Dasatinib or by inhibition of mitochondrial respiration was not sufficient to sustain survival and ATP levels in CLL samples sensitive to Dasatinib. The two subsets of primary CLL lymphocytes are characterized as well by a differential dependency on mitochondrial respiration and the use of anabolic or catabolic processes to cope with induced metabolic/energetic stress. Differential metabolic reprogramming between subsets is supported by the contrasting effect on the survival of Dasatinib treated CLL lymphocytes with pharmacological inhibition of two master metabolic regulators (mTorc1 and AMPK) as well as induced autophagy. Alternative metabolic organization between subsets is further supported by the differential basal expression (freshly purified lymphocytes) of active AMPK, regulators of glucose metabolism and modulators of AKT signaling. The contrasting metabolic features revealed by our strategy could be used to metabolically target CLL lymphocyte subsets creating new therapeutic windows for this disease for mTORC1 or AMPK inhibitors. Indeed, we report that Metformin, a drug used to treat diabetes was selectively cytotoxic to Dasatinib sensitive samples. Ultimately, we suggest that a similar strategy could be applied to other cancer types by using Dasatinib and/or relevant tyrosine kinase inhibitors.

## INTRODUCTION

Chronic lymphocytic leukemia (CLL), the most common adult B cell malignancy in the western world, is characterized by the accumulation of monoclonal peripheral (mature) CD5+ B cells in the blood and in primary and secondary lymphoid tissues of affected patients. A characteristic trait of these malignant lymphocytes is their low rate of proliferation, estimated to be a half of the proliferation rate of normal B-lymphocytes [1]. Prolonged survival of CLL lymphocytes has been associated with increased ligand-independent signaling activation downstream of the B-cell receptor. This has led to the clinical testing of non-receptor tyrosine kinase inhibitors that target key Src family kinases (i.e. Dasatinib), BTK family kinase inhibitors (i.e. Ibrutinib) and PI3K inhibitors (i.e. CAL-101) [2-4]. The activity of these tyrosine kinase inhibitors has been shown to be relatively independent of negative prognostic factors associated with the clinical response to the traditional therapeutic agents used to treat the disease such as a nucleoside analogue, fludarabine [5]. Poor outcome or prognostic factors in CLL are associated to the mutational status of the IgVH locus, the expression of CD38 and deletions at the ATM or p53 locus (del11q and del17p respectively) [6, 7].

It has been suggested that cancer cell metabolism is adapted to support high rates of proliferation and growth. These metabolic changes include higher rates of aerobic glycolysis (Warburg effect) and lipid metabolisms [8-10]. This knowledge has led to the evaluation of the effect of drugs used to treat metabolic disorders such as diabetes and obesity in the proliferation and survival of cancer cells [9, 11]. Preclinical studies in CLL suggest that interference with lipid metabolism is cytotoxic to these primary malignant cells *in vitro* [12-14]. However, the contribution of glucose metabolisms and, in particular, of aerobic glycolysis to primary CLL lymphocytes homeostasis is not known. In contrast to quiescent normal B lymphocytes [1, 15, 16], a recent metabolomic study suggests that CLL lymphocytes produce high levels of glycolytic intermediates and lactate, observations that are consistent with the Warburg effect [15].

In the past few years, various reports have shown that tyrosine kinase inhibitors (TKIs) including Dasatinib affect glucose metabolism ameliorating the fasting glucose level in diabetic and non diabetic individuals resulting in some cases in a successful treatment of cancer and diabetes simultaneously [17-20]. We have recently shown that Dasatinib can induce endoplasmic reticulum stress (ER stress) in primary CLL lymphocytes *in vitro* [21], a process known to induce glucose uptake and promote mitochondrial respiration [22, 23]. Furthermore, as shown by others in CML, we reported that inhibition of Dasatinib-induced autophagy in CLL results in sensitization to Dasatinib [21, 24]. For all the above, we hypothesize that resistance to Dasatinib is associated with a differential metabolic adaptation to metabolic stress in primary CLL lymphocytes. We report that the differences in the resistance to this TKI are associated with a lower sensitivity to mitochondrial complex I inhibition and the phosphorylation steady state of a master regulator of metabolism, the 5' AMP-activated protein kinase (AMPK). Our results demonstrate that Dasatinib-induces energetic stress in primary CLL lymphocytes *in vitro* and that AMPK activation contributes to Dasatinib-drug resistance in a subset of primary CLL samples independently of traditional prognostic factors.

## RESULTS

**Figure 1:**
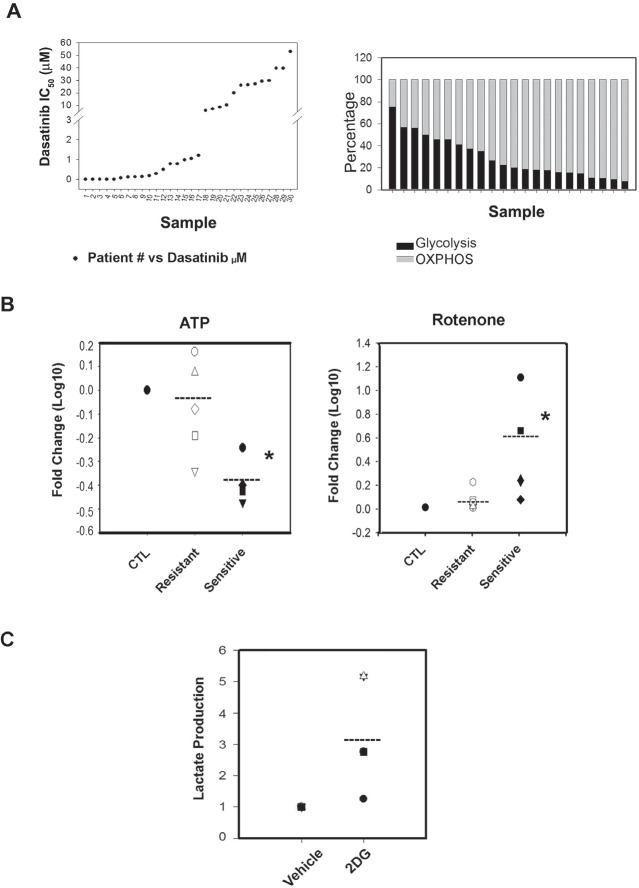
Analyzed samples and their bioenergetics' organization A.Left panel: Dasatinib IC50 distribution of 30 CLL patient's primary B-cell lymphocytes samples. We distinguished a set of Dasatinib sensitive (<0.1nM-1000nM) and a set of Dasatinib resistant samples (6-52μM). Right panel: The bioenergetics' profile of 22 samples of CLL primary lymphocytes cells. To calculate the Glycolysis (black boxes) and OXPHOS (grey boxes) level in the response to OXPHOS inhibitor, Oligomycin (2uM), we used lactate concentration ratios in the non treated/treated samples after 24h incubation. In high glucose media, Oligomycin increased significantly lactate production, 2.9 ± 0.5 folds (p=0.011) and OXPHOS contributes to the 70% in average to the bioenergetics need of the samples tested (t-Test p<0.001). The plotted differences in bioenergetics organization are not ordered following the samples' Dasatinib sensitivity. B.Left panel: Effect on ATP levels by Rotenone; Rotenone significantly decrease (*) ATP levels by 2.1 fold (Paired t-Test p<0.008) in Dasatinib sensitive samples (black shapes) vs. no effect (1.2 fold) on Dasatinib resistant samples (white shapes). The scatter plot shows fold changes in Log 10. Right panel: we assessed the effect of Rotenone at a toxic concentration of 500nM which induced cell death in sensitive and resistant samples nonetheless the effect was only significant (*) in sensitive samples (Paired t-Test p<0.001) (black shapes). C.Scatter plot showing that 2-DG affected by increasing lactate production in a set of representative Dasatinib sensitive and resistant samples (Dasatinib resistant samples-white shapes and sensitive samples-black shapes) and the values correspond to ratios fold differences (2-DG/Vehicle treatments).

We used primary CLL lymphocytes from 30 CLL cases followed at the Jewish General Hospital Hematology Clinic upon informed consent. Samples positive for Del17p and Del11q (deficient for p53 and ATM respectively) were excluded from this study. By their sensitivity to Dasatinib obtained by MTT essay, 17 of the samples fall in the sensitive range IC_50_s (1nM-1000nM) and 13 of the samples fall in the resistant range (6-52μM) (Table 1 and FIGURE 1A left panel). We confirmed Dasatinib IC_50_'s cytotoxicity by AnnexinV/PI staining and FSC/SSC analysis. There were no significant associations between the IgVH mutational status or CD38 expression and Dasatinib resistance (p>0.05).

**Table 1:** List of patients studied, their Dasatinib IC_50_, their glucose levels, RAI stage and clinical status as well as CD38 expression and IgVH mutational status

Patient #	RAI stage	Clinical status	CD38	IgVH	Dasatinib μM	Glucose
1	III	U	+	M	0.0	N
2	NA	T	−	U	0.0	N
3	II	U	−	M	0.0	N
4	IV	T	+	U	0.0	N
5	III	U	+	U	0.0	N
6	O	U	+	U	0.07	D
7	O	U	NA	M	0.10	H
8	IV	T	−	U	0.12	N
9	IV	U	−	M	0.13	H
10	IV	U	−	U	0.18	D
11	IV	T	−	U	0.28	N
12	NA	T	NA	M	0.50	H
13	II	T	−	U	0.78	N
14	III	U	+	M	0.78	N
15	III	U	−	M	0.97	D
16	IV	T	−	M	1.04	NA
17	IV	T	−	M	1.21	N
18	II	U	+	M	6.00	D
19	II	U	−	M	7.17	D
20	I	U	−	U	8.60	N
21	I	T	−	M	10.30	H
22	NA	U	NA	M	20.00	N
23	O	T	−	M	26.00	N
24	I	U	−	M	26.24	N
25	I	U	−	M	27.00	N
26	III	T	−	M	29.00	N
27	II	U	−	M	29.65	H
28	III	U	−	U	39.40	N
29	II	T	−	M	39.62	N
30	I	U	−	M	52.61	H

### Differential dependency on OXPHOS metabolisms in primary CLL lymphocytes *in vitro*

We first characterize the bioenergetics' profile of the samples and found no differences in basal (vehicle-treated) glucose consumption, lactate production or ATP levels between Dasatinib sensitive and Dasatinib resistant samples (data not shown). We next assessed the contribution of OXPHOS and glycolysis to the bioenergetics' of the samples as described before by the use of a non-toxic concentration of Oligomycin (2μM) an inhibitor of the mitochondrial complex V (ATP synthetase) [25]. We utilized the increase on lactate production (i.e. glycolytic capacity) induced by 2μM Oligomycin with respect to vehicle treated samples to estimate the contributions of OXPHOS to the bioenergetics of the samples tested. The results indicate that OXPHOS contributes to 70% on average to the bioenergetic needs of the samples tested (Figure 1A right panel, p<0.001) without differences between Dasatinib sensitive and resistant samples. We next tested the effect of a non toxic concentration of another inhibitor of OXPHOS, 200nM Rotenone which is a specific inhibitor of mitochondrial complex I [26] and resulted in a significant and similar increase in lactate production and glucose use as the one obtained by Oligomycin confirming the bioenergetic organization of CLL lymphocytes without differences between subsets. Nevertheless, Rotenone (200nM) showed a differential effect on ATP levels decreasing ATP levels by 2.1 fold in Dasatinib sensitive samples vs. 1.2 fold in Dasatinib resistant samples (p<0.008) (FIGURE 1B, left panel) [26]. Suggesting that in this subset of CLL lymphocytes, glycolysis might not be sufficient to compensate ATP production upon inhibition of mitochondrial respiration.

We confirm the higher dependency on OXPHOS of Dasatinib sensitive CLL samples by the use of a higher concentration of Rotenone. Rotenone, when used at 500nM induced cell death in all samples tested however, the effect was significant in sensitive samples (p<0.001) (FIGURE 1B right panel). There was however no significant differences in the fold changes of glucose use or lactate production between subsets induced by Rotenone when used either at 200nM or 500nM concentration (data not shown).

Our results indicate that in spite of comparable glycolytic capacity (i.e. increased rate of lactate production after OXPHOS inhibition), induction of glucose use and/or aerobic glycolysis was not sufficient to maintain ATP levels and viability in Dasatinib sensitive primary CLL lymphocytes.

### Differential dependency on OXPHOS metabolisms in primary CLL lymphocytes *in vitro* is abrogated by limiting glucose availability

**Figure 2:**
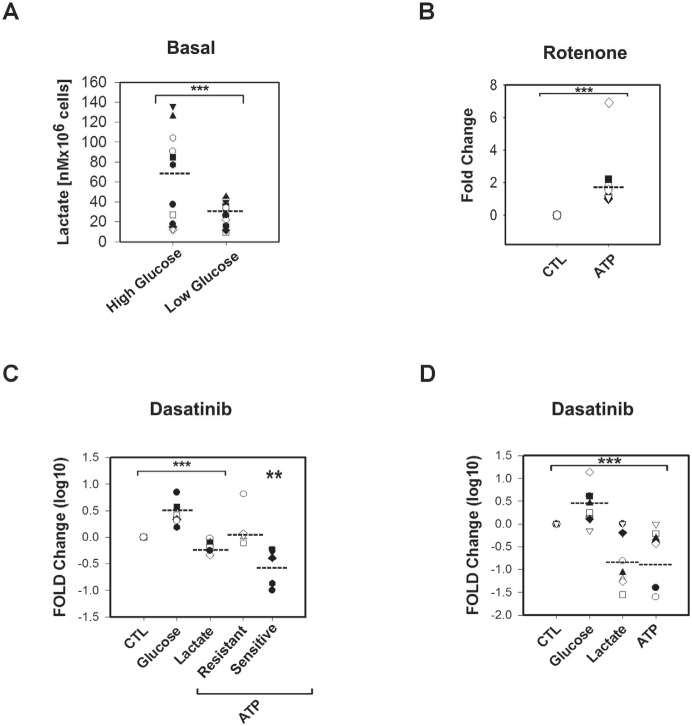
Metabolic differences between Dasatinib sensitive and resistant samples were abrogated in media with limited concentration of glucose (2mM) A.Scatter plot showing a significant lower (***) basal lactate production (nM per 106 cells) in 2mM glucose concentration media (t-Test p=0.023) than in higher glucose concentration media (10-12mM) (Dasatinib sensitive samples in black shapes while resistant are in white shapes). B.Scatter plots showing Rotenone effects on ATP levels. In low glucose concentration media (2mM) Rotenone induced significant (***) decreased in ATP levels (t-Test p<0.001) in both sets of samples (Dasatinib sensitive samples are black shapes, resistant are white shapes). C.Scatter plot showing Dasatinib IC50 effects fold changes in Log 10 on glucose uptake, lactate production and ATP levels in high glucose concentration media (Dasatinib/vehicle treatments). Dasatinib significantly (***) decreased lactate levels while significantly increased glucose uptake by 2 fold respect to paired vehicle-treated lymphocytes in both set of samples (t-Test p<0.001). At 24h after treatment a 4 fold decrease in ATP levels was significant (**) only in sensitive samples (black shapes) (Paired t-Test p=0.008). D.Scatter plot of significant (***) fold changes represented in Log 10, in 2mM glucose media showing that Dasatinib reduced significantly ATP levels in both resistant and sensitive samples by more than 2 fold (t-Test p=0.008) while inducing glucose use by 1.7 fold and reducing significantly lactate production by 3 fold to the same extent as in the case of conventional media.

We next assessed the effect of 2-Deoxy-D-glucose (2-DG), an inhibitor of glucose uptake, on the survival of primary CLL lymphocytes and found that 30μM-1mM range of 2-DG was not toxic to primary CLL lymphocytes *in vitro*. In addition, 2-DG induced AMPK phosphorylation *(Thr172)* (data not shown) as well as lactate production in both sensitive and resistant samples, although with a trend towards a higher increase in resistant samples suggesting that in primary CLL lymphocytes, as previously shown in other cell types, decreased glucose metabolisms can be compensated by glutaminolysis [27] (FIGURE 1C). Since glutaminolysis unlike glycolysis requires partial mitochondrial oxidation of glutamine derived glutamate, we anticipate that limiting glucose availability will increase the dependency of primary CLL lymphocytes to OXPHOS metabolism [28]. To test this, we cultured primary CLL samples in glucose limiting conditions (2mM). Although glucose consumption was not different than in regular media (p=0.26), the samples tested produced lower basal levels of lactate suggesting reduced glycolytic activity (FIGURE 2A). In this condition, 200nM Rotenone, in contrast to our observations in regular culture media, induced a comparable decrease in ATP in all samples tested regardless of their sensitivity to Dasatinib (FIGURE 2B). These results suggest that under limiting glucose availability, Dasatinib resistant primary CLL lymphocytes underwent a metabolic shift towards OXPHOS.

### Dasatinib induces glucose use and decreased lactate production in primary CLL lymphocytes independently of Dasatinib sensitivity

We next assessed the effect of the IC_50_ concentrations of Dasatinib on the bioenergetics of primary CLL lymphocytes. Twenty four hour treatment with the drug resulted in a significant decrease in lactate production while increasing glucose uptake by 2 fold with respect to paired vehicle-treated lymphocytes in both set of samples (p<0.001). In contrast, Dasatinib decreased ATP levels by 4 fold selectively in Dasatinib sensitive samples (p=0.008) (FIGURE 2C). However, Dasatinib treatment under limiting glucose availability (2mM) resulted in a comparable decrease in ATP levels in both Dasatinib sensitive and resistant samples by more than 2 fold (p<0.008) (FIGURE 2D). Dasatinib-induced glucose use seemed independent of glucose availability in the media. In contrast, the reduction on lactate production in 2mM glucose containing media was more irregular than in complete media (FIGURE 2CD). These results suggests that 1) Dasatinib in primary CLL lymphocytes induces energetic stress shifting glucose metabolisms from aerobic glycolysis and 2) decreased glucose availability and/or increased OXPHOS metabolisms sensitize primary CLL lymphocytes to Dasatinib *in vitro*.

### Differential expression of metabolic sensors and transducers in primary CLL lymphocytes

The distinctive responses in both sample sets to Dasatinib or Rotenone treatments encouraged us to assess for intrinsic differences in the expression/phosphorylation status of significant metabolic regulators. In freshly isolated CLL lymphocytes, we assess the expression of metabolic sensors such as LKB-1, AMPK, PPARa and PKM2 [14, 29, 30]; and metabolic transducers for instance ULK, mTor and Hif-1a [31]. In addition, we examined the expression levels of glucose transporters (GLUT-1 and GLUT-4), the mitochondrial uncoupling protein 2 (UCP2) as well as TIGAR fructose-2-6-bisphosphatase, a promoter of the pentose phosphate pathway, and CPT-1 (carnitine palmitoyltransferase-1), a transporter of fatty acid to the mitochondria.

PKM2, the isoenzyme responsible to catalyze the generation of pyruvate and ATP through glycolysis was expressed with no differences between subsets, suggesting a similar capacity to generate ATP through glycolysis [32-34]. The hypoxia inducible factor 1a (Hif-1a) was highly expressed in the samples. Of note, Hif-1a expression was significantly increased by 2 fold (range 1.2 to 7 fold, p=0.031) in primary cells cultivated in conventional culture media (10-12 mM glucose concentration RPMI1641 media), than the expression shown by freshly purified lymphocytes from whole blood samples suggesting higher stabilization if Hif-1a in conventional culture conditions (Supplementary Figure 1A). Stabilization of Hif-1a in normoxia conditions (21% oxygen) can be mediated by accumulation of TCA intermediates such as isocytrate, succinate and fumarate, three metabolites recently reported to be highly increased in primary CLL lymphocytes [15, 32-34].

**Figure 3:**
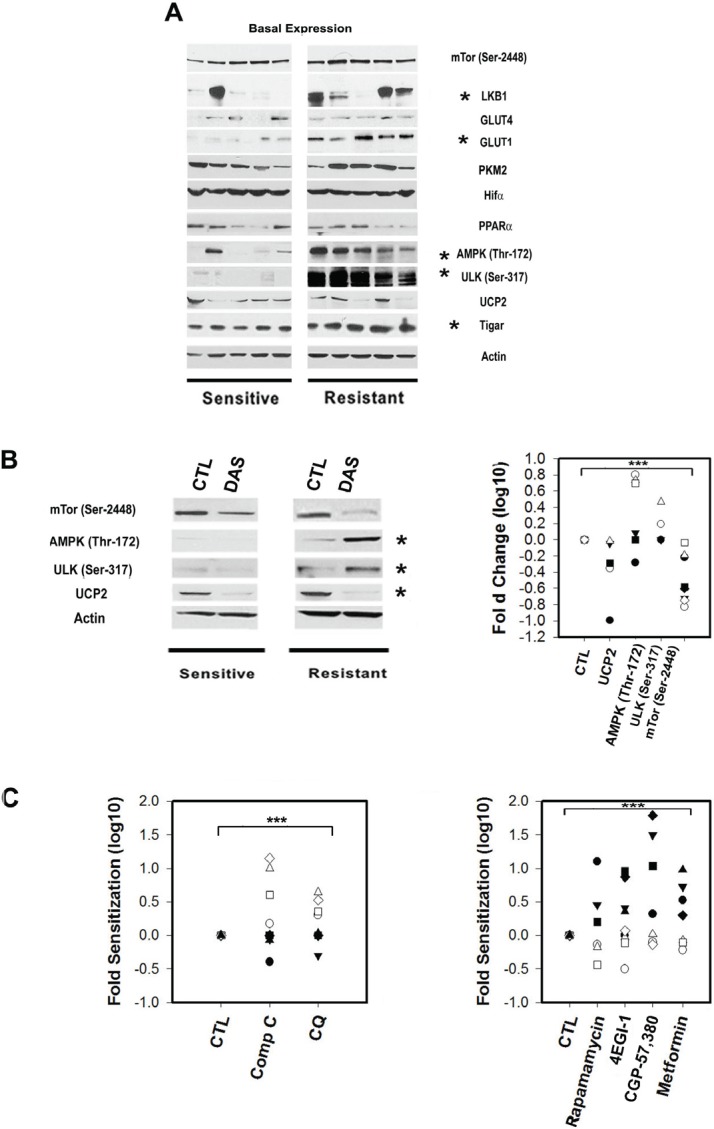
The expression and phosphorylation status of metabolic sensors and transducers in primary CLL lymphocytes is correlated with Dasatinib response A.Representative Western blots for Dasatinib sensitive and resistant samples showing significant differences (*) between sets for the expression of LKB1, activator of AMPK, was significantly decreased in sensitive samples (t-Test p=0.03) and correlated with Dasatinib in vitro resistance (IC50 values) (Spearman Corr. r=0.7, p= 0.03). Basal AMPK (Thr172) and ULK1/2 (Ser317) phosphorylation were significantly higher in resistant samples by 2 and 6.3 fold respectively (t-Test p=0.03 and p<0.001). Western blots analysis revealed similar expression of GLUT-4 in all samples tested and a significant lower expression of GLUT-1 in sensitive samples (Mann-Whitney U Statistic p=0.005). Resistant samples showed increased basal expression of TIGAR and was positively associated with Dasatinib resistance (Pearson Corr. r=0.75, p=0.02). PKM2, UCP2, PPARa, mTOR (Ser2448) and Hif-1a expression were variable and highly expressed in the samples tested but not associated with Dasatinib resistance. B.Left panel: Western blots showing Dasatinib in vitro treatment significantly (*) decreased mTor phosphorylation (Ser2448) and UCP2 protein levels by 5 fold (Paired t-Test p=0.004) and 2 fold (Paired t-Test p=0.008), respectively in all samples tested after 12h. While, Dasatinib-induced AMPKThr172/ULK1/2(Ser317) phosphorylation in Dasatinib resistant samples by 5 fold (Paired t-Test p=0.008), and 2.8 fold (Paired t-Test p=0.01). Right panel: scatter plot of the significant (***) effect in fold changes Log 10 of Dasatinib in Dasatinib sensitive (black shapes) and resistant samples (white shapes). C.Left panel: scatter plot showing ratios of co-treatment with Dasatinib and vehicle (CTL), 5μM compound C (Comp C) (an AMPK inhibitor) and 1μM Chloroquine (CQ) (an inhibitor of late autophagy). Both selective sensitized resistant samples (white shapes) to Dasatinib by 7 and 2 fold respectively (mean value) (Paired t-Test p<0.05). Right panel: scatter plot showing the effect on 4 drugs at non toxic concentrations on Dasatinib. Two mTORC1 inhibitors Rapamycin (100nM) and Metformin (1mM) as well as two downstream mTOR pathway inhibitors: CGP-57,380 (1μM) and 4EGI-1 (10μM) significantly (***) sensitized sensitive samples (black shapes) to Dasatinib with median sensitization values of 3.7, 5.3, 4.4 and 4.1 fold for Rapamycin, Metformin, CGP-57,380 and 4EGI-1 respectively (Paired t-Test p<0.01).

In addition, we found no significant differences in the expression of UCP2, PPARa and CPT-1 (FIGURE 3A and Supplementary FIGURE 1B). UCP2 expression in all CLL samples suggested that primary CLL lymphocytes upon ROS induction may have the capacity to increase proton conductance of mitochondrial inner membrane [35, 36] while the expression of CPT-1 and PPARa could indicate a high lipid metabolism capacity in primary CLL lymphocytes [14].

### The LKB/AMPK/ULK axis is constitutively active in Dasatinib resistant primary CLL lymphocytes:

We found that the expression of LKB1, an activator of AMPK, was significantly decreased in sensitive samples (p=0.03) and correlated with Dasatinib *in vitro* resistance (IC_50_ values) (r=0.7, p= 0.03). Statistical analysis revealed that in basal conditions, defined as freshly whole blood purified lymphocytes, AMPK (*Thr*172) and ULK1/2 (*Ser*317) phosphorylation (an AMPK downstream target required for the induction of autophagy) [30] were significantly higher in resistant samples when compared to sensitive samples by 2 and 6.3 fold respectively (p=0.03 and p<0.001, respectively). In addition AMPK (*Thr172)* and ULK1/2 (S*er317)* basal phosphorylation were significantly correlated to the response to Dasatinib (r=0.64 p=0.02 and r=0.83 p<0.0001, respectively) (FIGURE 3A). Furthermore, our results suggests that the differences in basal AMPK and ULK phosphorylation between subsets are intrinsic to the samples since there was no change on their expression by co-culturing primary CLL lymphocytes with stromal cells (BMS2 cells) previously reported to increase primary CLL lymphocyte survival by up-regulating survival pathways and affecting the metabolisms of these leukemic cells [37] (Supplementary FIGURE 1CD).

### The glucose transporter 1 (GLUT-1) and TIGAR are over-expressed in Dasatinib resistant samples:

Western blot analysis revealed similar expression of GLUT-4 in all samples tested but with a significant lower expression of GLUT-1 in sensitive samples (FIGURE 3A). This suggests that there is a differential regulation of glucose uptake between sensitive and resistant samples since GLUT-4 and GLUT-1 are distinctively regulated by PKC and PI3K respectively [38]. Differential capability for glucose use between subsets is also suggested by higher TIGAR expression in resistant samples (FIGURE 3A) which was positively associated with Dasatinib resistance (r=0.75, p=0.02). TIGAR has a fructose-2,6-bisphosphate activity and has been shown to decrease glycolysis by shifting the use of glucose to the pentose phosphate pathway for NADPH generation and reduction of ROS [39].

### The PI3K/AKT/mTORC1 axis is differentially regulated in primary CLL lymphocytes:

In contrast to the differences in AMPK and ULK1/2 phosphorylation, mTor (*Ser2448*) basal phosphorylation was detected in all samples tested (FIGURE 3A). Consistently with constitutive mTORC1 activation, phosphorylation of P70S6K (*Thr*389) and 4E-BP1 (*Thr*37/46) were detectable in almost all of the samples tested (Supplementary Figure 1E). However, a differential regulation of the PI3K-AKT axis between sets is suggested by the down-regulation of the basal expression of a negative regulator of AKT (PTEN) (three-fold, P<0.001), and the up-regulation of the positive regulator of AKT (TCL-1) (three-fold p=0.04) in sensitive samples while a divergent upstream regulation of the PI3K pathway between subsets was suggested by the significantly higher phosphorylation of the insulin receptor substrate 1 (IRS-1) (*Thr*307/312) in resistant samples with respect to sensitive samples (p=0.012) (Supplementary FIGURE 1E).

### Differential contribution of the mTORC1 and AMPK checkpoints to the metabolic homeostasis in primary CLL lymphocytes *in vitro* under metabolic stress

**Figure 4:**
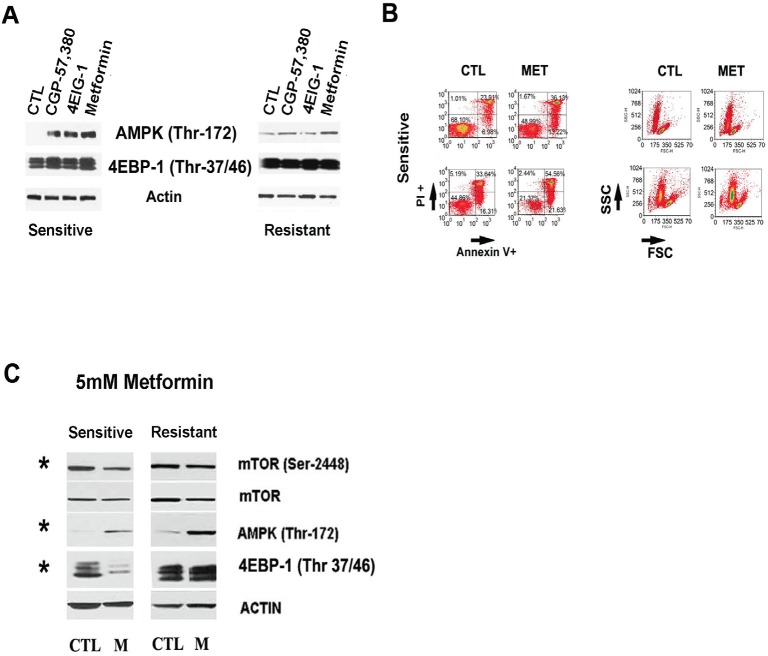
Complex I and downstream mTor pathway in Dasatinib sensitization A.Western blots showing that non toxic doses of Metformin (1mM), CGP-57,380 (1μM), Rapamycin (100nM) and 4EGI-1(10μM) increased AMPK (Thr172) in sensitive samples while they did not affecte 4EBP-1 (Thr37/46) phosphorylation, an mTor downstream target. B.Annexin V/PI staining and FSC-SSC assay showing 5mM Metformin effect after 48h treatment. The graphs are for representative samples and show that Metformin at 5mM induced cell death and apoptosis which were selectively in sensitive samples (t-Test p=0.035). C.Western blots showing 5mM Metformin effect on mTor and AMPK pathway. A higher concentration of 5mM Metformin treatments at 12h increased significantly (*) AMPK (Thr172) in resistant and sensitive samples together with a decrease in mTor-2448 in both sets however the inhibition of mTOR (Ser2448) as well as 4EBP-1 (Thr37/46) phosphorylation was significantly decreased (*) in sensitive samples compared to resistant samples by 2 folds for mTor (Paired t-Test p=0.029) and more than 3 folds in the case of 4EBP-1 (Paired t-Test p=0.011).

We next assessed the effect of Dasatinib on significant targets twelve hours after Dasatinib treatment with equally cytotoxic concentrations meaning that each sample was treated with its own IC_50_. In this condition, *in vitro* treatment with the drug resulted in decreased mTor phosphorylation (*Ser2448*) and UCP2 protein levels by 5 fold (p=0.004) and 2 fold (p=0.008), respectively in all samples tested (FIGURE 3B, left panel). The decreased levels of UCP2 suggest a drug-induced increase in mitochondrial membrane potential. This was supported by Dasatinib induction of increased mitochondrial biomass staining with Mitotracker green in all the tested samples (Supplementary FIGURE 1F). Additionally, Dasatinib-induced AMPK and ULK phosphorylation in resistant samples by 5 fold (p=0.008), and 2.8 fold (p=0.01), respectively (FIGURE 3B, left panel and right panel). The results suggest that there are differences in regulation of the AMPK-mTor metabolic checkpoint between subsets. Thus, we assess the effect of non toxic concentrations of inhibitors of mTorc1 signaling or AMPK as well as autophagy in the resistance to Dasatinib in both subsets. In light of our results showing a differential dependency on OXPHOS between subsets, we also assess the effect of Metformin. Metformin is a partial mitochondrial complex I inhibitor used to treat diabetes [11, 40]. Co-treatment of Dasatinib with specific inhibitors of AMPK, mTORC1 signaling or late autophagy resulted in clearly differential response between subsets. We found that the combinations of Dasatinib with 5μM compound C (an AMPK inhibitor) or Chloroquine (1μM) (an inhibitor of late autophagy) selectively sensitized resistant samples to Dasatinib by 7 and 2 fold respectively (mean value) (p<0.05) (FIGURE 3C, left panel). In contrast, sensitive samples were sensitized to Dasatinib by Metformin (1mM) in 5.3 fold (median value) (p=0.004) (FIGURE 3C, right panel) or by other inhibitors of mTORC1 signaling pathway. We used Rapamycin as well as two downstream mTORC1 pathway inhibitors: CGP-57380 a MAP-kinase interacting kinase-1 (Mnk1, MNK1) inhibitor and a hinder of eIF4E phosphorylation [41] and the 4EGI-1, a polypeptide that inhibits cap-dependent protein translation by disruption of the eIF4E/eIF4G association [42]. The median sensitization values we obtained for Dasatinib sensitive samples were of 3.7 for Rapamycin, 4.4 for CGP-57380 and 4.1 fold for 4EGI-1 (p<0.05) (FIGURE 3C, right panel). Interestingly, the concentrations of CGP-57380 (1μM), 4EGI-1 (10μM) or Metformin (1mM) although not toxic induced AMPK (*Thr*172) phosphorylation selectively in sensitive samples (FIGURE 4A), supporting a higher dependency of sensitive samples on eIF4E mediated functions to maintain bioenergetics' homeostasis. Of note CGP-57380, 4EGI-1 or Metformin when used alone did not affect 4EBP-1 phosphorylation, suggesting that the sensitization effect of the drugs is not mediated by direct inhibition of mTorc1 (FIGURE 4A).

### Metformin targets Dasatinib sensitive samples *in vitro*

The higher dependency of Dasatinib sensitive samples of OXPHOS and mTorc1 activity under Dasatinib-induced stress anticipated that Metformin would be selectively cytotoxic to Dasatinib sensitive samples. Metformin, in addition to inhibiting mitochondrial complex I can inhibit mTorc1 activity both directly and indirectly through activation of AMPK [11, 43-45]. Indeed, we found that 5mM Metformin treatment decreased cell size and induced variable apoptosis selectively in sensitive samples (FIGURE 4B). Western blot analysis 12 hours after 5mM Metformin treatment resulted in increased phosphorylation AMPK (*Thr*172) while decreasing phosphorylation mTor (*Ser*2448) in all samples tested (Figure 4C). However, inhibition of mTor (*Ser*2448) phosphorylation as well as 4EBP-1 was significantly higher in sensitive samples (two-fold, p=0.029) (FIGURE 4C). Together, Metformin and Rotenone effects on sensitive samples supported a dependency of sensitive samples not only on mTor pathway but also on complex I for their survival.

## DISCUSSION

Cancer cells, unlike their normal counterparts, exhibit significantly different and higher metabolic requirements; cancer cells consume additional nutrients due to their high rate proliferation profile thus it is thought that cancer cells may have to rearrange their metabolic pathways to compensate their higher need for ATP and synthesis intermediates. Principally, the Warburg effect reinstates how cancer cells would supplement their metabolic requirements [46, 47]. It has been shown that cancer cells exhibit heterogeneous metabolic rewiring beyond the Warburg effect suggesting that this heterogeneity is a manifestation of different metabolic adaptations by cancer cells. However, in agreement with Warburg's initial hypothesis evidence exists that addiction to glycolysis can be driven by OXPHOS dysfunction. For examples, in lung and melanoma cells inhibition of glucose uptake resulted in forced OXPHOS as in this condition mitochondria were unable to maintain ATP production [48]. In these tumor cells glycolysis was shown to be driven by mutated BRAF activity, resulting to addiction to this oncogene. In addition, the metabolisms of cancer cells can be driven by tumor associated cells. In breast cancer for example, glycolysis carried by tumour associated cells results in the release into the tumor microenvironment of metabolite that induce OXPHOS and mitochondrial biogenesis in the adjacent cancer cells [49, 50].

Regarding CLL primary cells, attempts have been made to characterize their metabolic organization. The seminal studies regarding CLL metabolism suggested that compared to normal lymphocytes, CLL lymphocytes consumed less glucose in a similar manner than lymphocytes from patients with diabetes mellitus [46, 47, 51, 52]. In contrast to these early studies, a recent metabolomic study using freshly isolated CLL lymphocytes revealed that these malignant lymphocytes display high levels of aerobic glycolysis and produce lactate which are consistent with the use of glucose and the Warburg effect [53]. In addition we found evidence that micro-environmental modifications affected the metabolism of primary CLL lymphocytes by the changes in Hif-1a expression between freshly purified lymphocytes and the ones grown in culture media. Hif-1a expression in primary CLL lymphocytes in normoxia conditions have been previously reported and was associated with decreased degradation [32], however stabilization of Hif-1a expression has also been shown to be mediated as well by increased ROS and accumulation of mitochondrial metabolites [34]. Among Hif-1a functions of promoting glycolysis, it restricts the mitochondria oxidation of glucose derived pyruvate [34]. Nevertheless, more studies should be conducted on CLL metabolic changes between cultured and freshly isolated lymphocytes to understand through which mechanism Hif-1a is stabilized since hydroxylase activity could be inhibited not only by low concentrations of O_2_ but as well by high concentrations of tricarboxylic acid cycle intermediates (isocitrate, oxaloacetate, succinate, or fumarate), or chelators of Fe(II) changes [32-34].

Regarding CLL bioenergetics organization, we found that *in vitro*, primary CLL lymphocytes use significant amounts of glucose and produce lactate. However, in spite of similar glycolytic capacity, Dasatinib sensitive samples were more dependent on OXPHOS to maintain ATP levels than Dasatinib resistant samples. However, a shift towards OXPHOS metabolism mediated by the use of limited glucose availability abrogated this difference. In a similar manner, the more pronounced decrease on ATP levels induced by Dasatinib in sensitive samples was abrogated by decreasing the concentration of glucose in the culture media. Since Dasatinib decreased lactate production, it is possible that this TKI decreases glycolysis driven by tonic activation of the B cell receptor, as shown in lymphoma cell lines [54]. Together, our results suggest that Dasatinib sensitive samples are more sensitive to both inhibition of OXPHOS and glycolysis than Dasatinib resistant samples and that this difference might be associated with a higher capacity in this later subset of samples to adapt to energetic stress. Together with decreased lactate production, Dasatinib increased glucose use, suggesting that the drug induces energetic stress as well as a shift in glucose use in primary CLL lymphocytes. Our results suggest that Dasatinib induces OXPHOS since increased mitochondrial biogenesis was observed in both sensitive and resistant samples. The observed effect of the drug on UCP2 expression levels suggests an adaptation to increased OXPHOS with elevated mitochondrial membrane potential. In fact, it has been reported that the genetic loss of UCP2 leads to a faster proliferative rate associated with increased glucose metabolism [35, 36, 55]. In addition, it is possible that Dasatinib-induced stress increases the use of glucose through the pentose phosphate pathway (PPP). This pathway generates reduced glutathione (GSH) from NADPH, a major ROS scavenger, reportedly abundantly present in CLL primary samples [53]. In keeping with the reported induction of ROS by Dasatinib, up-regulation of GSH production would be an expected homeostatic adaptation [56]. Furthermore, Dasatinib has been reported to disrupt the mitochondrial proton transport complex [57]. Here, in particular, complex I seemed to be involved directly in sensitive samples metabolic response and the higher cytotoxic effect of Dasatinib. Higher glucose metabolism and glucose dependency in Dasatinib resistant samples is suggested by the observation that they express higher levels of the glucose transporter GLUT-1 and by the immediate effect on Dasatinib and Rotenone resistance in these samples by the reduction of glucose availability. In agreement with the higher glucose metabolism in the Dasatinib resistant samples we found higher levels of phosphorylation of the IGFR1 target, IRS-1 in this subset. Thus, Dasatinib resistant samples in contrast to Dasatinib sensitive samples could sustain glucose flux through the autocrine activation of the IGFR1 and PKC-AMPK [58, 59]. The high expression levels of CPT-1 and PPARa we have observed in CLL indicates, as was suggested by others, that lipid metabolism is important for these malignant lymphocytes [14]. Inhibition of fatty acid metabolism has been shown to be cytotoxic to primary CLL lymphocytes *in vitro* and sensitize them to glucocorticoids. As shown for CLL and Acute-Lymphoblastic-Leukemia (ALL), glucocorticoids are being used as therapeutic agents [60] are known to inhibit glycolysis and have an effect on GLUT-1 emphasizing the targeting of glycolytic metabolism as therapeutic strategy and suggesting that Dasatinib resistant samples might be more sensitive to glucocorticoids than Dasatinib sensitive samples [14, 61, 62].

As a result of the present work, further studies are required to assess the role of fatty acid metabolism in Dasatinib resistance and the relation with differential re-wiring of metabolic route. However, higher basal rates of fatty acid oxidation would be expected in Dasatinib resistant samples driven by AMPK activation and IGFR1 signaling [63, 64]. In these settings, the decreased levels of UCP2 induced by Dasatinib treatment might as well reflect a shift from lipid to carbohydrate metabolisms mirroring the effects reported by abrogation UCP2 expression, which results in a shift from carbohydrate to lipid metabolism [35, 36, 55].

A shift or adjustment of the importance of fatty acid metabolism in CLL seemed to also happen after Dasatinib treatment and could be supported by the effect of ABT-737, a BH3 mimetic compound which targets BCL-XL, BCL-2, and BCL-W [65]. It has been reported that CLL resistance to drug treatment is associated to high expression of Bcl2, a protein directly involved in fatty acid oxidation (FAO) by its interaction with CPT-1 [66, 67]. The sensitization we found to Dasatinib by ABT-737 suggests that Dasatinib as a metabolic stressor not only affects glycolysis it may also affect FAO metabolism and open a therapeutic window where a Bcl2 inhibitor could be efficient to promote apoptosis in CLL (Supplementary FIGURE 2).

The differential metabolic organization between sets can be supported by differences in the induction of other biological processes by Dasatinib treatments. We have previously reported that Dasatinib induced endoplasmic reticulum stress as well as autophagy on wild type p53 samples, furthermore we indicated a synergistic effect of pifithrin-a, 3-MA or Chloroquine on Dasatinib cytotoxicity in p53 proficient CLL cells [68]. Here the two established subpopulations are p53 proficient CLL cells and we found that endoplasmic reticulum stress as well as ROS were induced by the drug only in Dasatinib resistant cells (Supplementary FIGURE 3) though neither the use of Salubrinal, an inhibitor of eIF2a dephosphorylation and ER stress-mediated apoptosis, nor NAC, a ROS scavenger, affected Dasatinib sensitivity (data not shown) suggesting that these metabolic features, although not associated with Dasatinib resistance might be a distinctive response to energetic stress among subsets.

In addition, in sensitive samples (as we previously reported in p53 deficient CLL lymphocytes) Dasatinib failed to induce AMPK phosphorylation or autophagy at lower nM concentrations (IC_50_s) or clinical achievable concentrations (180nM) (data not shown). These results, together with the sensitization effect of Chloroquine or compound C on Dasatinib sensitivity suggests that unlike induction ER stress or ROS, autophagy and AMPK activation contribute to the restoration of metabolic homeostasis after Dasatinib treatment in the resistant set. In light of our results, we predict that alternative metabolic rewiring and response to metabolic stress would be expected in primary CLL lymphocytes with deficient p53 (del17p) or ATM (del11q) signaling. Since both p53 and ATM are involved in the regulation of glucose use through glycolysis and the pentose phosphate pathway as well as mitochondrial function [69, 70].

As AMPK activation and autophagy can serve as protective mechanisms conferring resistance to growth factor deprivation and maintenance of proliferative quiescence [71]; we speculate that the differential basal activation of AMPK and ULK among CLL subsets is associated with differential dependency of anabolic or catabolic processes to the maintenance of homeostasis and survival under energetic/metabolic stress. AMPK has been shown to repress anabolic processes (i.e. lipid and protein synthesis) while promoting catabolic processes (i.e. fatty acid oxidation and autophagy) under energetic stress and for the maintenance of proliferative quiescence [30, 72, 73]. In this scenario, Dasatinib resistant samples would be more dependent on catabolic processes driven by AMPK while sensitive samples would rely on anabolic processes driven by mTorc1 signaling to maintain homeostasis.

## METHODS

### Patient samples

Peripheral blood samples from 30 patients with a diagnosis of B-CLL attending the clinic at the Jewish General Hospital were obtained with the patients' informed consent in accordance with the ethical approval granted by the Jewish General Hospital Research Ethics Committee. A total of 40% (12 of 30) of patients had previously received treatment. Therapies given were Chlorambucil, Fludarabine, FC (Fludarabine and Cyclophosphamide), FCR (Fludarabine, Cyclophosphamide, Rituximab), CHOP (Cyclophosphamide, Doxorubicin, Vincristine, and Prednisolone), and Alemtuzumab. Clinical stage at diagnosis was based on the RAI clinical staging system (Table 1).

### Glucose uptake and Lactate production measurements, calculation of metabolic organization

The experiments were performed in two different culture medias, conventional media used to culture CLL lymphocytes *in vitro* and media containing physiological concentrations of glucose hereafter, high glucose -12mM, and low glucose-2mM, respectively.

The culture medium, where a million cells were treated with vehicle or as indicated, was collected after 24 hr and analyzed for glucose and lactate concentrations. For D-lactate production we used D-Lactate Colorimetric Assay Kit (BioVision or Sciencell) following the manufacturer instructions. A colorimetric assay, of coupling glucose oxidase and horseradish peroxidase was used to measure glucose consumption in accordance with Blake and Mc Lean's protocol [78]. All reagents were obtained from Sigma-Aldrich. Briefly, 10uL of each culture media sample was added to a 500uL reaction mixture containing 4-aminoantipyrine, N-ethyl-N-sulfopropyl-m-toluidine in a sodium phosphate buffer to which was added horseradish peroxidase and ddH_2_O. Glucose oxidase stock was then added to start the reaction which was measured 1 hour later for absorbance at 562nm. Resulting glucose levels were compared to values obtained for complete RPMI 1640 media conditioned without cells to determine glucose consumption. The results were normalized to the number of viable cells in each condition.

To calculate the glycolysis and OXPHOS level in the response to two OXPHOS inhibitors such as Oligomycin and Rotenone, we used the previously reported formula [25]: Lac(c), lactate concentration in the control medium/non treated samples after 24 hr incubation; Lac(o), lactate concentration in the medium after 24 hr of incubation with 2uM Oligomycin and 200nM Rotenone. The glycolysis and OXPHOS % were then calculated as Lac(c)/Lac(o) *100 and 100-Glycolysis %, respectively.

### ATP levels measurements

Cellular ATP changes were measured by the bioluminescence ATP determination Kit reagent according to the instructions (Molecular Probes^TM^). After 24 hrs treatments the 600,000 cells were collected and counted by hemocytometer.

### Cytotoxicity assay-MTT essay

Lymphocytes were isolated from the peripheral blood using Ficoll-Hypaque purification as described previously [79-82]. The CLL-lymphocyte population was composed of 97.7±2.1% (expressed as the mean %±S.D.) malignant B lymphocytes. The MTT (3-(4,5-dimethyl-2-thiazolyl)-2,5-diphenyl-2*H*-tetrazolium bromide) assay was performed 72 hrs after treatment as described in Amrein et al [21]. 20μl MTT solution at 5mg/ml in 1X dPBS was added into each well and incubated for 3 hours at 37°C and 5% CO_2_. Mitochondrial dehydrogenases of viable cells reduced the water-soluble MTT to water-insoluble formazan crystals, which were solubilized with 25 μl Sorensen solution and 100 μl of DMSO. The effect of Dasatinib (Bristol-Myers Squibb, New Brunswick, NJ, USA) was described as IC_50_ (concentration resulting in 50% of control). The cytotoxicity of other drugs on Dasatinib was evaluated as the ratio between Dasatinib IC_50_ when used alone over Dasatinib IC_50_ in the presence of different inhibitors used. Table 2 shows median of these ratios and concentrations of each drug used.

### Expression and activation of protein by flow cytometry analysis

One million cells were treated per assay; they were fixed with 1% formaldehyde followed by permeabilization in methanol. We employed Cell Signaling anti- AMPKa (*Thr*172) (rabbit mAB #2535), anti-ULK1 (*Ser* 317) (rabbit mAB#6887) and anti-mTOR (*Ser* 2448) (rabbit mAB#2971). The phospho-specific antibodies were labeled with Alexa Fluor488 goat anti-rabbit (Invitrogen-Molecular Probe A11034) used as secondary antibody and positive cells were visualized with a FACSCalibur flow cytometer (BD Biosciences, San Jose, CA) equipped with CellQuest software (BD Biosciences). For CD38 characterization we used Human CD38 APC conjudated (Invitrogen MHCD3805) and as isotype Mouse IgG1 APC conjugated (BD Biosciences#340442).

### Expression of proteins by Western blot

Aliquots of CLL lymphocytes (3 to 10 × 10^7^cells) were treated with whole-cell lysis buffer (10 mM Tris-HCl, 250 mM sodium chloride, 50 mM sodium fluoride, 0.5% Triton X-100, 0.1% sodium dodecyl sulfate, 10% glycerol, 1 complete pill of protease inhibitor mixture (Roche), 1 mM phenylmethysulfonyl fluoride, 100μM sodium orthovandate, 2mM iodoacetic acid, and 5 mM ZnCl_2_). Protein concentration was determined by Thermo Scientific Pierce BCA Protein Assay Kit following supplier stipulations. Each sample (30μg protein) was separated on a 4% to 12% Bis-Tris precast acrylamide gel (BioRad) and transferred to nitrocellulose membrane (BioRad Laboratories, Hercules, CA, USA). Western blots were probed with antibodies against the following antibodies: from Santa Cruz Biotechnology Bcl2, c-ABL, Mcl-1, UCP2 (C-20), PPARa (H-98), Glut-1, Glut-4 and Bip/GRP78 (Santa Cruz Biotechnology, Santa Cruz, CA, USA); from Cell Signaling we used Raptor, Rictor, AMPK (Thr172), ULK1/2(Ser317), TCL1, 4EBP-1, 4EBP-1 (Thr37/46), mTOR, mTOR (Ser2448), p70S6K (Thr 389), eIF4E (Ser209), MNK1 (Thr197/202); from Novus Syk (Tyr348) (Novus, Cedarlane); TIGAR was from Abcam and LC3 was from Abgen. Hif1a was from Cayman Chemical and CPT-1C was from Proteintech^TM^. The secondary antibodies were horseradish peroxidase–conjugated anti-mouse (GE Healthcare Bio-Sciences Corp, Piscataway, NJ, USA), anti-rabbit (KPL Inc., Gaithersburg, MD, USA) or anti-goat (Santa Cruz) and detection was performed by an enhanced chemiluminescence method (Immobilon Western, Millipore). The signals were analyzed using ImagesJ 1.44p (Wayne Rasband, NIH) and normalized to Actin (goat mAB SC-1616, Santa Cruz).

### Apoptosis-PI/annexin V staining

Cells were analyzed for Annexin V binding (APC- BD) and propidium iodide (PI- Sigma-Aldrich) incorporation to distinguish between apoptotic and necrotic cells. Apoptosis was analyzed using the following manufacturer's instructions. In brief, the cells were harvested at 24 and 48 hrs, washed with 1X dPBS, and incubated for 15 min in 1X binding buffer containing Annexin V and PI. Before analysis by flow cytometry, the cell suspension was diluted 1:4 of 1X binding buffer. We used a FACSCalibur flow cytometer (BD Biosciences, San Jose, CA) equipped with CellQuest software (BD Biosciences).

### Acridine orange (AO) and LC3 conversion: Autophagy detection

Autophagy induction was monitored by the detection of acidic vesicular organelles (AVOs), which characterized late autophagy, using the FACScan flow cytometer and CellQuest software by staining the cells with acridine orange (1μg/ml) for 15 min after different drug treatments. Red (FL3-protonated AO) and green (FL1-AO outside acidic compartments) filters were used to detect autophagy. The induction of autophagy was also assessed by detecting a specific marker of autophagy, the increase of autophagosomal membrane form of microtubule-associated protein light chain 3 (LC3I-II) at protein level by Western Blot.

### Drug and compounds

Dasatinib was used alone or in combination. We assay sensitization on Dasatinib IC_50_s of CGP 57,380 (1 to 10μM), 3-Methyladenine (3-MA) (300uM), Chloroquine (5μM), Rapamycin (100nM), and 2-DG (60μM to 1mM) were purchased from Sigma-Aldrich; 4EGI-1 (10μM) was purchased from Santa Cruz. Metformin was assayed in 1mM to 5mM and was obtained from Sigma Aldrich as well as Oligomycin (2μM) and the complex I inhibitor, Rotenone. Rotenone was assayed in 200nM and 500nM a concentration reported as enough to decrease ATP and block oxygen respiration [26].

To study the effect of ROS production on Dasatinib response cells were pretreated with 5mM of the anti-oxidant N-acetyl-l-cysteine (NAC, Sigma, Taufkirchen, Germany) and combined with Dasatinib IC_50_ for 24 hours.

In addition a BH3 mimetic drug, ABT-737 (Selleck Chemicals), which target BCL-XL, BCL-2, and BCL-W was used in 1 nM and was purchased from Cedarlane, Canada.

### DCF –ROS detection and mitochondrial biomass

2′-7′-Dichlorodihydrofluorescein diacetate (DCFH-DA) is one of the most widely used techniques for directly measuring the redox state of a cell. The cell-permeant H_2_DCF-DA enters the cell where intracellular esterases cleave off the diacetate group. The resulting H_2_DCF is retained in the cytoplasm and oxidized to the highly fluorescent 2',7'-dichlorofluorescein (DCF) DCF by ROS which can be measured by flow cytometry. One millon cells were treated with Dasatinib IC_50_ for 1, 2, 4, 6 and 12 hrs. Cells were then incubated with 25mg/ml 2′-7′-dichlorofluorescin diacetate (DCFH-DA- Invitrogen D-399) for 30 minutes at 37°C for the detection of intracellular peroxides (mainly H_2_O_2_). Cells were collected and washed in 1X dPBS. Fluorescence was measured by flow cytometry and analyzed using FCS Express v3.0.

Mitochondrial biomass after vehicle or Dasatinib IC_50_ 12 hrs treatments was determined by the use of 25nM Mitotracker® Green FM (Molecular Probes-Invitrogen) and visualized by flow cytometry (FL1).

### Co-Culture of CLL Lymphocytes

Stromal cells co-cultured effect on B-CLL cell was assessed briefly as follow, CLL lymphocytes were co-cultured with BMS2, a murine bone marrow stromal cell line (BM-derived, support clone 2, [83] in 1:20 ratio and different drug and combinations were tested.

### Statistical analysis

Statistical analysis was performed by parametric and non-parametric analysis (2-tailed *t* test, , Pearson and Spearman Rank correlation analysis and *pair*-*t*-test). All tests were performed using Sigma Stat/Plot software (Systat Software Inc., San Jose, CA, USA). Differences between groups or associations were considered significant if p<0.05.

## CONCLUSIONS

It has been proposed that cancer cells can achieve metabolic homeostasis by either increasing their metabolic activity and ensuring metabolic fluxes by the maintenance of anabolic processes or by reducing their metabolic demands promoting catabolic processes [10, 74, 75]. It is possible that CLL lymphocytes use distinctively anabolic or catabolic strategies. The induction of catabolism and the increased activation of anti-anabolic processes are mainly supported in resistant samples which showed an increased autophagy and ER stress [76] two catabolic processes increased after Dasatinib treatment only in resistant samples while sensitive samples primarily seemed to rely on mTORC1 signaling (Supplementary Figure 3). The differential basal activation of AMPK and ULK indeed suggest differential setting of the AMPK/mTor metabolic checkpoint. Of interest, differences in AMPK activation reported here were observed in freshly isolated samples and did not change by co-incubation with stromal cells, suggesting that this feature is intrinsic to the samples. The use of Dasatinib reveals this intrinsic differential metabolic rewiring among CLL lymphocytes. This is further supported by the reliance on the maintenance of anabolic (i.e. mTor signaling in sensitive samples) or catabolic processes (i.e. AMPK and autophagy) in response to Dasatinib-induced metabolic stress. This feature could be used to target these two subsets of CLL lymphocytes using other TKIs. Indeed we find in CLL samples without cytogenetic aberrations such as del11q and del17p, that Dasatinib resistance correlated with the resistance to a pan-phosphoinositide 3-kinase (PI3K) inhibitor, BKM120 (r=0.52, p<0.001) (data not shown) [77]. As well, Dasatinib resistant samples might be sensitive to IGFR-1 inhibitors.

## Supplementary Figures


