# Supplementary material for: Resistance to Dasatinib in primary chronic lymphocytic leukemia lymphocytes involves AMPK-mediated energetic re-programming
Source: Oncotarget. 2013 Nov 7;4(12):2550–66. doi: 10.18632/oncotarget.1508 (PMC3926848; doi:10.18632/oncotarget.1508)
Supplement: Supplementary file 1 [file oncotarget-04-2550-s001.pdf]

# Resistance to Dasatinib in primary chronic lymphocytic leukemia lymphocytes involves AMPK-mediated energetic re-programming - Marignac et al

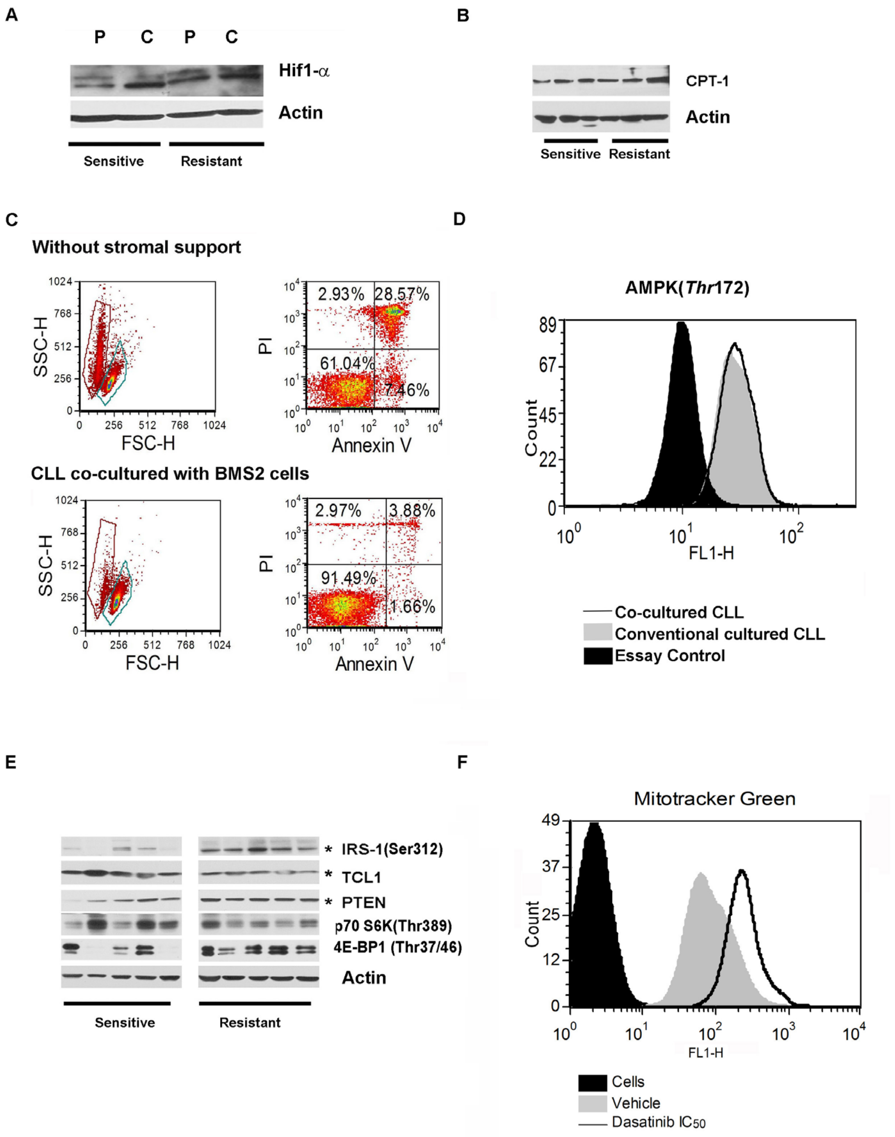


**Supplementary Figure 1: Differential setting for CLL Dasatinib sensitivity subpopulations.** A. Western blot of 2 representative Dasatinib resistant and sensitive samples showing that Hif-1a expression was stabilized in CLL samples. Hif-1a was significantly increased by 2 fold (range 1.2 to 7 fold, Paired t-Test p=0.031) in primary cells cultivated in conventional culture media (C) compared to the expression shown by freshly purified lymphocytes (P). B. Western blot showing in CLL representative samples that CPT-1, was similarly expressed across the samples. C. We assessed the effect of the murine bone marrow stromal cell line (BMS2) on CLL. The graph shows the FSC-SSC (left panels) and Annexin V/PI staining (right panels) analysis in a representative CLL sample. Co-culture decreased the rate of spontaneous death of primary CLL lymphocytes represented by decreased late apoptosis (red gate-death cells). D. FACScan flow cytometric analysis showing that co-culture with stromal cells (black line) did not affect the basal activation of AMPK(*Thr*172) observed in the samples cultured in conventional media (grey filling/line), here we show a representative Dasatinib resistant sample. E. Constitutive mTORC1 activation and a differential regulation of PI3K-AKT axis between sets. Western blots showing 5 representative Dasatinib sensitive and resistant samples. The phosphorylation of P70S6K (*Thr*389) and 4EBP-1 (*Thr*37/46) were detectable in almost all of the samples tested. In Dasatinib resistant samples the basal expression of a negative regulator of AKT (PTEN) was significantly (*) up-regulated (three-fold, t-Test p<0.001) together with the down-regulation of the positive regulator of AKT (TCL1) (three-fold, t-Test p=0.04) compared to Dasatinib sensitive samples. IRS-1 (*Thr*307/312) was significantly (*) phosphorylated in Dasatinib resistant samples (t-Test p=0.012). F. Flow cytometry histogram of Mitotracker® Green FM (Molecular Probes) in a representative sample showing Dasatinib $IC_{50}$ induction (black line) of increased mitochondrial biomass staining compared to vehicle (grey filling/line).

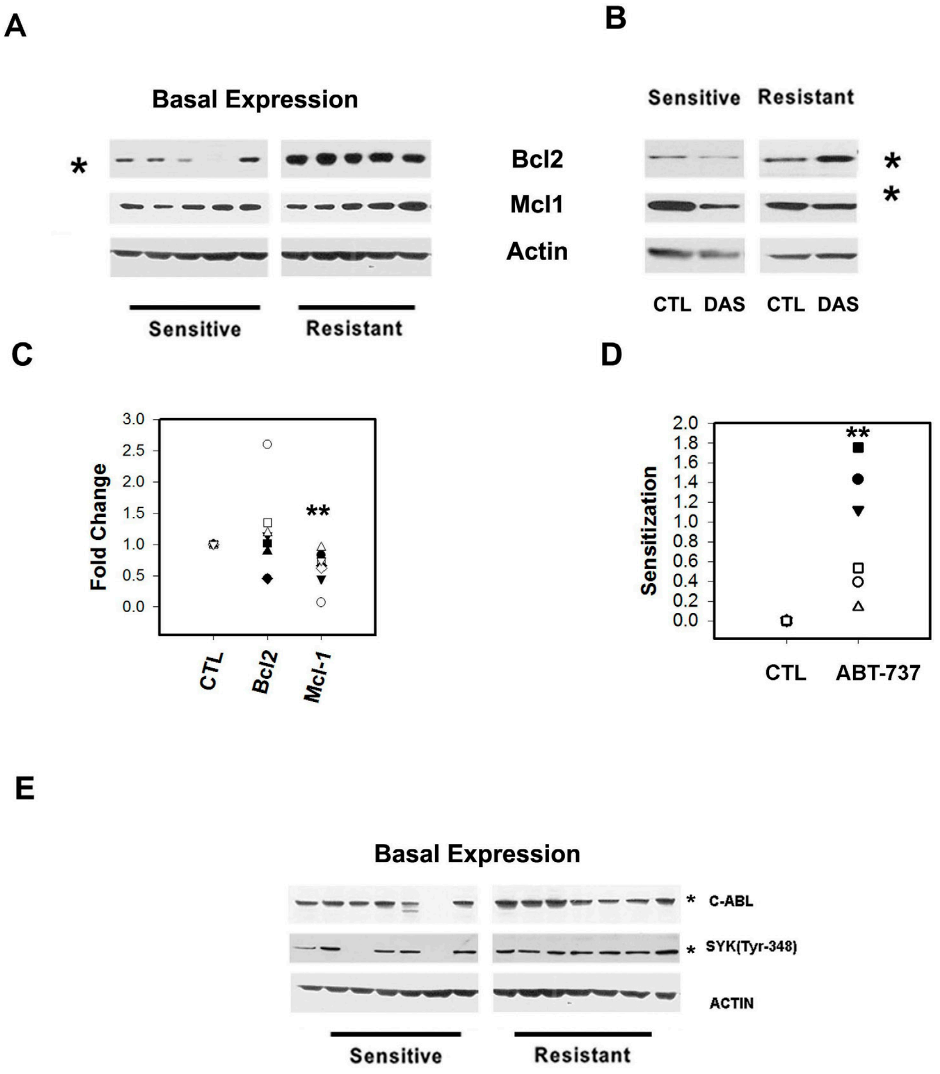


**Supplementary Figure 2: Bcl2 inhibition sensitized CLL samples to TKI, Dasatinib.** A. Western blot analysis showing that Bcl2 protein was expressed in all the samples and was significantly (*) higher in Dasatinib resistant samples compared to sensitive samples (t-Test $p<0.05$). Another member of bcl2 family, Mcl1, did not show differences between sets. B and C. Western blot and graph showing the effect of *in vitro* Dasatinib $IC_{50}$ administration (DAS) on Bcl2 and Mcl1 protein expression levels. Bcl2 significantly (*) increased by 2 fold only in resistant samples (white shapes) (Paired t-Test $p<0.05$) while Mcl1 significantly decreased in all samples tested (Paired t-Test $p=0.002$) compared to the vehicle treated samples (CTL). D. Scatter plot showing that simultaneous *in vitro* co-treatment with Dasatinib and the BH3 mimetic ABT-737, which target BCL-XL, BCL-2, and BCL-W [65] affected significantly (**) Dasatinib cytotoxicity in both resistant and sensitive samples (Paired t-Test $p<0.014$) and synergized Dasatinib cytotoxicity by average of 2 fold in 75% of the resistant samples tested (Paired t-Test $p=0.023$) (black shapes) and by more than 3 fold in 60% of the sensitive samples tested (Paired t-Test $p=0.072$) (black shapes). E. Western blot showing expression in 6 representative samples of each Dasatinib response subpopulations. Syk phosphorylation and c-ABL expression were significantly higher (*) in Dasatinib resistant samples as compared to sensitive samples (t-Test $p=0.019$ and $p=0.002$, respectively).

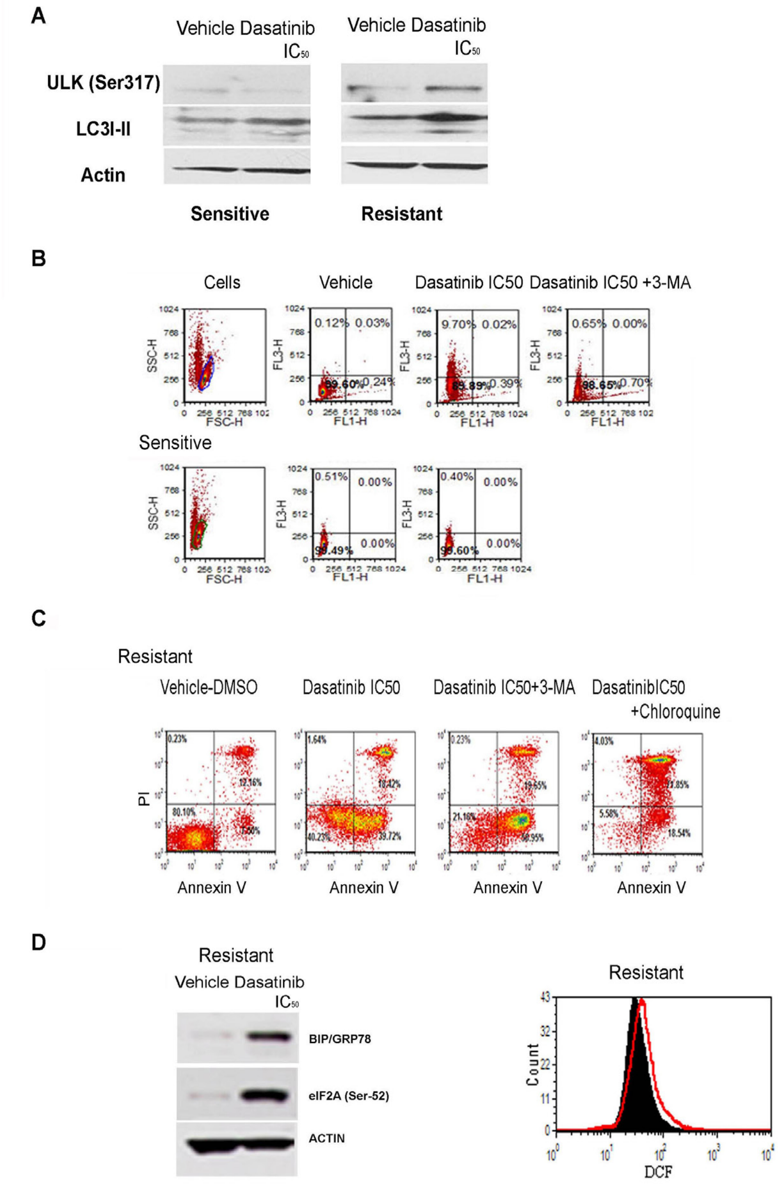


**Supplementary Figure 3: The differential metabolic organization between sets can be evidenced by differences in drug-induced biological processes.** A. Representative Western blots showing Dasatinib inducing the increased of ULK1/2 (*Ser*317) and the conversion of LC3 I to II in Dasatinib resistant samples. B. Pharmacological induction of autophagy by Dasatinib in Dasatinib resistant samples. Dasatinib $IC_{50}$ treated cells were stained with acridine orange (1µg/ml). The induction of autophagy was measured by FACScan flow cytometry analysis by FL1-H, green color intensity; and FL3-H, red color intensity. Top of the grid (FL3-H increased) was considered as AVOs. Quantification of the development of AVO was expressed by the percentage of total cells. Sensitive samples lack the induction of autophagy not only at Dasatinib $IC_{50}$ concentration but at 10 fold higher concentrations (180nM). The confirmation of autophagic AVOs was performed by the co-treatment with 3-MA of Dasatinib resistant samples which showed a decreased of positive cells for FL3-H. C. FACScan flow cytometry analysis of AV and PI staining showed that 300µM 3-MA and 5µM Chloroquine, inhibitors of early and late autophagy respectively, significantly sensitized Dasatinib resistant cells to Dasatinib and increased the percentage of positive cells for AV/PI by four-fold, (t-Test $p<0.001$). D. Left panel: ER stress showed by the increased of ER stress hallmark protein expression. As shown, Dasatinib induced phosphorylation of eIF2a (*Ser*52) more than four-fold (Paired t-Test $p<0.01$) and increased the expression of BIP/GRP78 more than three-fold in resistant samples (Paired t-Test p= 0.009). No significant changes for these two targets were observed in sensitive samples (data not shown). Right panel: ROS were determined by flow cytometry using DCFH-DA (Invitrogen). The effect of Dasatinib on ROS production was analyzed at 1, 2, 4, 6 and 12h. Resistant samples showed an increase of DCF positive cells at 4h of Dasatinib $IC_{50}$ treatments (Red line) compared with vehicle treated samples (black filling/line) (range 11-22%) (Paired t-Test p=0.002) and no change was observed in sensitive samples at any of the assayed time points (data not shown).
